# Supplementary material for: Codon usage biases co-evolve with transcription termination machinery to suppress premature cleavage and polyadenylation
Source: eLife. 2018 Mar 16;7:e33569. doi: 10.7554/eLife.33569 (PMC5869017; doi:10.7554/eLife.33569)
Supplement: Supplementary file 1. [file elife-33569-supp1.docx]

**Supplemental file 1**

| strain | Genotype | Origin/reference |
| --- | --- | --- |
| 4200 | *a* | FGSC #4200 |
| 87-3 | *a* | (1) |
| 301-6 | *his-3 a* | (1) |
| 303-3 | *frq::hph+; his-3; a* | (1) |
| 1 | *frq::hph+; his3*+::pKAJ120 | (2) |
| 2 | *frq::hph+; his3*+::*frq*-deopt1 | This study |
| 3 | *frq::hph+; his3*+::*frq*-deopt2 | This study |
| 4 | *frq::hph+; his3*+:: pKAJ120.aq | (3) |
| 5 | *frq::hph+; his3*+::*frq*-deopt2.aq | This study |
| 6 | *frq::hph+; his3*+::f-*frq* | (2) |
| 7 | *frq::hph+; his3*+::*frq*-deopt3 | This study |
| 8 | *frq::hph+; his3*+::*frq*-deopt4 | This study |
| 9 | *frq::hph+; his3*+::*frq*-deopt5 | This study |
| 10 | *frq::hph+; his3*+::*frq*-deopt6 | This study |
| 11 | *frq::hph+; his3*+::*frq*-deopt7 | This study |
| 12 | *frq::hph+; his3*+::*frq*-deopt4* | This study |
| 13 | *his3*+:: pBM61.*qa-2-gfp*-*NCU09435*-wt | This study |
| 14 | *his3*+:: pBM61.*qa-2-gfp*-*NCU09435*-opt | This study |
| 15 | *his3*+:: pBM61.*qa-2-gfp*-*NCU02034*-wt | This study |
| 16 | *his3*+:: pBM61.*qa-2-gfp*-*NCU02034*-deopt | This study |
|  |  |  |
|  |  |  |

1. Cha, J., Yuan, H. and Liu, Y. (2011) Regulation of the activity and cellular localization of the circadian clock protein FRQ. *Journal of Biological Chemistry*, **286**, 11469-­‐11478.

2. Zhou, M., Guo, J., Cha, J., Chae, M., Chen, S., Barral, J.M., Sachs, M.S. and Liu, Y. (2013) Non-­‐optimal codon usage affects expression, structure and function of clock protein FRQ. *Nature*, **495**, 111-­‐115.

3. Xue, Z., Ye, Q., Anson, S.R., Yang, J., Xiao, G., Kowbel, D., Glass, N.L., Crosthwaite, S.K. and Liu, Y. (2014) Transcriptional interference by antisense RNA is required for circadian clock function. *Nature*, **514**, 650.
