# Supplementary material for: Codon usage biases co-evolve with transcription termination machinery to suppress premature cleavage and polyadenylation
Source: eLife. 2018 Mar 16;7:e33569. doi: 10.7554/eLife.33569 (PMC5869017; doi:10.7554/eLife.33569)
Supplement: Supplementary file 2. [file elife-33569-supp2.docx]

# Supplemental file 2

**Primers used for 2P-seq:**

Reverse transcription primer for 2P-seq:

5′-(Phos)-GATCGGAAGAGCGTCGTGTAGGGAAAGAGTGTAGATCTCGGTG GTCGC-(SpC18)-CACTCA-(SpC18)-TTCAGACGTGTGCTCTTCCGATCTATTG ATGGTGCCTACAG-3′

Forward library PCR primer:

5′-AATGATACGGCGACCACCGAGATCTACAC-3′

Reverse library PCR primer: Index 6 Primer for Illumina:

5´-CAAGCAGAAGACGGCATACGAGAT-ATTGGCGTGACTGGAGTTCAGAC GTGTGCTCTTCCGATC-s-T-3´

Index 12 Primer for Illumina:

5´-CAAGCAGAAGACGGCATACGAGAT-ATTGGCGTGACTGGAGTTCAGAC GTGTGCTCTTCCGATC-s-T-3´

# Supplemental file 2 continued primers used for qPCR:

| Primer | Sequence 5’−> 3’ |
| --- | --- |
| Fig 2D 1F | GCAGAGGACCCTGAACTTT |
| Fig 2D 1R | TGCTCTCTTGCTCACTTTCC |
| Fig 2D 2F | CCAGTCCCCTCGTCCCCTCCTGGC |
| Fig 2D 2R | GTCAGGTGAGTGCGGGTGCGGGTG |
| Fig 2D 3F | GAGGAACCAGAACGTAGCAG |
| Fig 2D 3R | GCAGGATAAACGGAGAAATGAC |
| Fig 2D 4F | GGACACCTTTCATTACAAACCG |
| Fig 2D 4R | TCCGCTAAAATCCCACTTCG |
| Fig 2D 5F | GATACCGAGACTGATGTGCG |
| Fig 2D 5R | AGCATGTCCACCTCTTTTCC |
| ψ63 F | CCCTAGACCTCTAAGGAATTGGA |
| ψ63 R | ACGATCCCGCGGTAGTTATTA |
| Fig S2A 1F | CTGGGACGTAACTGGTAGAAAC |
| Fig S2A 1R | AGGCACAAGAGCCCATTATAC |
| Fig S2A 2F | GGATCTACATCCGTCTCAACAC |
| Fig S2A 2R | GGTGAAGGAGCAATGGTATGA |
| Fig S2A control F | ATAACTTCGTCTTCGGCCAG |
| Fig S2A control R | ACATCGAGAACCTGGTCAAC |
